# Phorbol ester activates human mesenchymal stem cells to inhibit B cells and ameliorate lupus symptoms in MRL.*Fas*^lpr^ mice

**DOI:** 10.7150/thno.46835

**Published:** 2020-08-13

**Authors:** Hong Kyung Lee, Hyung Sook Kim, Minji Pyo, Eun Jae Park, Sundong Jang, Hye Won Jun, Tae Yong Lee, Kyung Suk Kim, Sang-Cheol Bae, Youngsoo Kim, Jin Tae Hong, Jaesuk Yun, Sang-Bae Han

**Affiliations:** 1 College of Pharmacy, Chungbuk National University, Cheongju, Chungbuk 28160, Republic of Korea.; 2 Bioengineering Institute, Corestem Inc., Gyeonggi 13486, Republic of Korea.; 3 Hanyang University Hospital for Rheumatic Diseases, Seoul 04763, Republic of Korea.

**Keywords:** B cell, CXCL10, mesenchymal stem cell, PD-L1, phorbol ester, systemic lupus erythematosus

## Abstract

**Rationale:** Systemic lupus erythematosus (SLE) is a multi-organ autoimmune disease characterized by autoantibody production by hyper-activated B cells. Although mesenchymal stem cells (MSCs) ameliorate lupus symptoms by inhibiting T cells, whether they inhibit B cells has been controversial. Here we address this issue and reveal how to prime MSCs to inhibit B cells and improve the efficacy of MSCs in SLE.

**Methods:** We examined the effect of MSCs on purified B cells *in vitro* and the therapeutic efficacy of MSCs in lupus-prone MRL.*Fas*^lpr^ mice. We screened chemicals for their ability to activate MSCs to inhibit B cells.

**Results:** Mouse bone marrow-derived MSCs inhibited mouse B cells in a CXCL12-dependent manner, whereas human bone marrow-derived MSCs (hMSCs) did not inhibit human B (hB) cells. We used a chemical approach to overcome this hurdle and found that phorbol myristate acetate (PMA), phorbol 12,13-dibutyrate, and ingenol-3-angelate rendered hMSCs capable of inhibiting IgM production by hB cells. As to the mechanism, PMA-primed hMSCs attracted hB cells in a CXCL10-dependent manner and induced hB cell apoptosis in a PD-L1-dependent manner. Finally, we showed that PMA-primed hMSCs were better than naïve hMSCs at ameliorating SLE progression in MRL.*Fas*^lpr^ mice.

**Conclusion:** Taken together, our data demonstrate that phorbol esters might be good tool compounds to activate MSCs to inhibit B cells and suggest that our chemical approach might allow for improvements in the therapeutic efficacy of hMSCs in SLE.

## Introduction

Systemic lupus erythematosus (SLE) is an autoimmune disease characterized by the production of autoantibodies to ubiquitous self-antigens 1. Although various abnormalities of immune cells have been implicated in SLE pathogenesis, excessive B cell activation seems to play a crucial role 2. Mesenchymal stem cells (MSCs) are multipotent adult stem cells that have emerged as a promising therapy for the treatment of SLE. Adoptive transfer of MSCs to lupus-prone MRL*.Fas*^lpr^ mice increased their survival and decreased anti-dsDNA antibody level and nephritis 3-9. In clinical studies, MSCs improved renal functions and decreased autoantibody production 10-15. As to the underlying mechanisms, MSCs inhibit T cell functions by producing such soluble mediators as IL-10, nitric oxide (NO), tumor growth factor (TGF)-β, prostaglandin E_2_ (PGE_2_), and indoleamine 2,3-dioxygenase (IDO) 16-18. In addition, MSCs suppress T cell functions by CCL2- and Fas-dependent contact inhibition 19, 20. MSCs also inhibit the functions of dendritic cells, neutrophils, and natural killer cells, but enhance those of regulatory T cells 21-24. However, whether MSCs inhibit B cells has been controversial. Some studies have shown that MSCs inhibit proliferation, antibody and cytokine production, and migration of B cells 25-28. But others reported that MSCs cannot inhibit the proliferation of and antibody production by B cells 29, 30, and even enhance B cell functions 23, 31. Reportedly, MSCs indirectly inhibit B cell proliferation through the inhibition of T cells 29, 32.

Since B cells play a crucial role in SLE progression, it is essential to understand the effect of MSCs on B cells. In this study, we first investigated whether mouse and human MSCs inhibited B cells or not. We found that mouse MSCs (mMSCs) inhibited mouse B (mB) cells, but human MSCs (hMSCs) did not inhibit human B (hB) cells, suggesting a species difference in MSC effects on B cells. Then, we extended the scope of our study to find chemicals that could improve hMSC functions. We found that phorbol esters activated hMSCs to inhibit hB cells and enhanced the therapeutic activity of hMSCs in a SLE mouse model. We suggest here that a chemical approach might be useful to generate clinically useful hMSCs for the treatment of SLE patients.

## Materials and Methods

### Preparation of MSCs

hMSCs were generated from bone marrow (BM) cells aspirated from the posterior iliac crest of healthy donors. Mononuclear cells were collected by density gradient centrifugation (Ficoll-Paque; GE Healthcare Bio-Sciences AB, Uppsala, Sweden) and were cultured at 2 × 10^7^ cells/T175 flask in CSBM-A06 medium (Corestem Inc., Gyeonggi, Korea) containing 10% fetal bovine serum (BD Biosciences, Franklin Lakes, NJ, USA), 2.5 mM L-glutamine, and penicillin/streptomycin (WelGene, Gyeonggi, Korea) in a 5% CO_2_ incubator at 37 °C. Medium was changed every 3-4 days and non-adherent cells were removed. Adherent cells were sub-cultured on day 10 or 11 (passage 1). hMSCs were used in experiments at passages 3-5 33. The surface marker profile of hMSCs was CD29^+^CD44^+^CD73^+^CD105^+^CD90^+^CD34^-^CD45^-^HLA-DR^-^ (data not shown). All hMSC studies were approved by the Institutional Review Board of Hanyang University Hospital and were carried out in accordance with the approved guidelines. All participants provided written informed consent.

mMSCs were generated from the BM cells of tibiae and femurs of 6-8-week-old C3H/HeN mice (Orient Bio, Gyeonggi, Korea). Red blood cells were lysed with ACK buffer, and BM cells were cultured at 1 × 10^7^ cells/well of a 6-well plate in α-MEM medium containing 10% fetal bovine serum, 2 mM L-glutamine, and penicillin/streptomycin in a 5% CO_2_ incubator at 37 °C. Medium was changed every 3 days and non-adherent cells were removed. Adherent cells were sub-cultured on day 10 or 11 (passage 1) and used in experiments at day 17-21 33. The surface marker profile of mMSCs was Sca-1^+^CD44^+^CD73^+^CD45^-^CD11b^-^CD11c^-^Gr-1^-^MHC-II^-^ (data not shown). All animal studies were approved by the Chungbuk National University Animal Experimentation Ethics Committee and were carried out in accordance with the approved guidelines.

### Priming of hMSCs with chemicals

hMSCs were treated with phorbol myristate acetate (PMA; 10 ng/mL) for 24 h, washed three times with medium, and used immediately in experiments. We also treated hMSCs with various chemicals for 24 h at the concentrations indicated in Table S1.

### Preparation of B cells and mitogen assay

Human peripheral blood mononuclear cells were donated by the Chungbuk Red Cross Blood Center (Cheongju, Korea). Lymphocytes were isolated from these cells by density gradient centrifugation (Ficoll-Paque) 34. hB cells were isolated from lymphocytes using a human B cell isolation kit (Miltenyi Biotec, Auburn, CA, USA). mB cells were purified from spleen cells of MRL*.Fas*^lpr^ mice by a negative depletion method using mouse B cell isolation kit (Miltenyi Biotec) 9. Purity of hB and mB cells was typically >90%. B cells (1 × 10^5^ cells/well) and MSCs (0.01-0.1 × 10^5^ cells/well) were added in 200 µL to the wells of 96-well plates. CpG-oligodeoxynucleotide (ODN; 5 μg/mL) was used to activate hB cells, and lipopolysaccharide (LPS; 1 μg/mL) was used to activate mB cells. To measure B cell proliferation, cells were pulsed with [^3^H]-thymidine (113 Ci/nmol; NEN, Boston, MA, USA) at a concentration of 1 μCi/well for the last 18 h and were harvested on day 3 using an automated cell harvester (Inotech, Dottikon, Switzerland). The amount of [^3^H]-thymidine incorporated into the cells was measured using a Wallac Microbeta scintillation counter (Wallac, Turku, Finland) 3.

### Time-lapse imaging and transwell assay

For time-lapse imaging, dishes were pre-warmed for 24 h in a 5% CO_2_ incubator at 37 °C. MSCs (70 µL of 0.3 × 10^6^ cells/mL) were seeded into the left chamber and B cells (70 µL of 1 × 10^6^ cells/mL) into the right chamber of culture-insert µ-Dish^35mm^ culture dishes (ibidi GmbH, Martinsried, Germany). Cell-containing dishes were incubated for 3 h under the microscope and then inserts were carefully removed. Time-lapse imaging was performed with a Biostation IM-Q microscope (Nikon, Tokyo, Japan) equipped with a 10× magnification objective (numeric aperture 0.5) and an environmental chamber kept at 37 °C and 5% CO_2_. Images were acquired every 2 min for 12 h and were analyzed by using Imaris software 9.3.0 (Oxford Instruments plc, Abingdon, UK). The number of migrating B cells was counted 4.

For transwell assay, B cells (1 × 10^6^ cells/mL) were stained with 5-chloromethylfluorescein diacetate (CMFDA; 2 µM) at 37 °C for 15 min and washed three times with serum-free medium. B cells (1 × 10^5^ cells; 100 µL) were added to each upper well of transwell plates with a 5-µm insert (Corning, Corning, NY, USA). Various concentrations of chemokines or MSCs were added to the lower wells in 600 µL of complete RPMI 1640 medium. The number of B cells migrated to the lower well over 1.5 h was counted using a flow cytometer (FACSCalibur; BD Biosciences) 35. In some experiments, B cells were pre-incubated with the CCR2 antagonist RS102895 (30 µg/mL, Sigma-Aldrich, St. Louis, MO, USA) 36 or CXCL12 antagonist AMD3100 (300 µg/mL, Sigma-Aldrich) 37 for 1 h.

### Apoptosis assay

hB cells (1 × 10^6^ cells) and hMSCs (0.1 × 10^6^ cells) were added in 2 mL onto 35-mm culture dishes (BD Biosciences) and cultured for 24 h. hB cell apoptosis was determined in three ways. First, hB cells were stained with anti-CD19 antibody conjugated with APC and then with FITC-annexin and propidium iodide for 15 min (FITC-Annexin V Apoptosis Detection Kit, BD Biosciences). hB cells were analyzed using a flow cytometer (FACSCalibur) and the data were processed using Cell Quest Pro software (BD Biosciences) 38. Second, hB cells were stained with anti-CD19 antibody conjugated with APC and then labeled with FITC-ApoStat (Intracellular Caspase Detection ApoStat kit, Bio-Techne, Minneapolis, MN, USA) for 1 h. hB cells were analyzed using a flow cytometer (FACSCalibur) and the data were processed using Cell Quest Pro software 39. Third, CellEvent Caspase-3/7 Green ReadyProbes Reagent (Thermo Fisher Scientific, Carlsbad, CA, USA) was directly added to the co-culture of hB cells and hMSCs 40. Time-lapse imaging was performed with a Biostation IM-Q microscope (Nikon) and images were acquired in two channels (phase contrast and green filter) every 10 min for 24 h 4. Images were analyzed by using Imaris software 9.3.0. Green-fluorescent cells were considered apoptotic.

### Lupus-prone MRL.*Fas*^lpr^ mouse model

MRL*.*MpJ-*Tnfrsf6^Faslpr^*/J (called MRL.*Fas*^lpr^ hereafter) mice lack *Fas* and spontaneously develop an SLE-like disease 20. The onset and symptom severity in these mice depend on their genetic background. Female MRL*.Fas*^lpr^ mice die at an average age of 17 weeks and males at 22 weeks. Similar to SLE patients, MRL*.Fas*^lpr^ mice have a marked increase in anti-dsDNA antibodies in their blood and develop severe nephritis. Female MRL*.Fas*^lpr^ mice were purchased from the Jackson Laboratory (Bar Harbor, ME, USA). Mice were housed in specific pathogen-free conditions at 21-24 °C and 40-60% relative humidity under a 12 h light/dark cycle and randomized into 3 groups. Mice were injected intravenously with PBS (vehicle, n = 6), 4 × 10^4^ naïve hMSCs/mouse (n = 6), or 4 × 10^4^ PMA-hMSCs/mouse (n = 6) once at the age of 12 weeks. Survival rate and body weight were examined every 2 weeks. Serum were collected every 3 weeks and stored at -70 °C until used. The levels of anti-dsDNA IgG and total IgG in serum and the levels of protein in urine were measured by using ELISA kits purchased from Alpha Diagnostic International (San Antonio, TX, USA), eBioscience (San Diego, CA, USA), and Sigma-Aldrich, respectively, according to the manufacturers' instructions.

### Immunohistochemistry (IHC)

Kidneys were isolated from the surviving MRL.*Fas*^lpr^ mice at 22 weeks of age, fixed with 4% formalin, and immersed in PBS 5. After dehydration with ethanol and xylene, the tissues were embedded in paraffin and cut into 4-µm sections. After removing paraffin, sections were hydrated and heated in a microwave oven (650 W, 20 min) for antigen retrieval, after which endogenous peroxidase activity was blocked with 3% hydrogen peroxide. To detect immune cells in the kidney, sections were incubated with the following primary goat antibodies against mouse IgG (diluted 1:100; Jackson ImmunoResearch, West Grove, PA, USA), C3 complement (1:100; GeneTex, San Antonio, TX, USA), CD19 (1:100; BioLegend, San Diego, CA, USA), CD3 (1:100; Santa Cruz Biotechnology, Dallas, TX, USA), F4/80 (1:100; Santa Cruz Biotechnology), CD209b (1:100; Santa Cruz Biotechnology), and Foxp3 (1:50; Abcam, Cambridge, UK) at 4 °C overnight. Then all sections were incubated with secondary antibody (anti-goat IgG conjugated with horseradish peroxidase; Vector Laboratories, Burlingame, CA, USA) for 1 h at room temperature. Signals were developed with a two-component high-sensitivity diaminobenzidine chromogenic substrate (Vector Laboratories) for 10 min and the sections were counter-stained with hematoxylin. Stained area (%) was calculated with ImageJ software (NIH, Bethesda, MD, USA) as follows: IHC stained area (brown staining) / total area (brown + non-brown staining).

### RNA interference

Small interfering RNAs (siRNAs) were purchased from Bioneer (Daejon, Korea). Their sequences are shown in Table S2. MSCs were transfected with 100 nM siRNAs using Lipofectamine RNAiMAX reagent (Thermo Fisher Scientific) following the manufacturer's protocol. Cells were incubated at 37 °C in a CO_2_ incubator for 48 h 41.

### RT-PCR, ELISA, and nitric oxide (NO) assay

Total RNA was isolated from MSCs using TRIZOL Reagent (Thermo Fisher Scientific). RNA was quantified using a spectrophotometer and stored at -80 °C at a concentration of 1 mg/mL. cDNA was synthesized from 3 µg total RNA using an RT kit (Bioneer). PCR was used to examine the levels of mRNAs of chemokines, cytokines, and other proteins in spleen cells of MRL.*Fas*^lpr^ mice at 22 weeks of age. The primer sequences are shown in Table S3. PCR products were separated on 1% agarose gels and stained with 5 µg/mL ethidium bromide 33.

The levels of CCL2, CCL4, CCL5, CXCL10, CXCL12, IFN-γ, IL-10, PGE_2_, and TGF-β, accumulated in medium were measured by ELISA (Bio-Techne). The levels of IDO were measured by ELISA (BlueGene Biotech; Shanghai, China) 20. The levels of IgM and IgG were determined by ELISA (Thermo Fisher Scientific). The level of nitrite accumulation in medium was used as an indicator NO production 43. Briefly, the cell culture supernatants were mixed with an equal volume of Griess reagent (1% sulfanilamide, 0.1% naphthylethyl-enediamine dihydrochloride, and 2% phosphoric acid) and incubated at room temperature for 10 min. Nitrite concentrations were measured as optical density at 540 nm.

### Western blotting

Cells were lysed in ice-cold cell lysis buffer (Cell Signaling Technology, Danvers, MA, USA; 20 mM Tris-HCl, pH 7.5; 150 mM NaCl; 1 mM Na_2_EDTA; 1 mM EGTA; 1% Triton X-100; 2.5 mM sodium pyrophosphate; 1 mM β-glycerophosphate; 1 mM Na_3_VO_4_; 1 µg/mL leupeptin). Proteins were separated by 10% SDS-PAGE and transferred to a PDVF membrane (MilliporeSigma, Burlington, MA, USA). Membrane was blocked with 5% skim milk in TBS/Tween-20 (TTBS) for 1 h and incubated with primary antibody in TTBS containing 5% BSA overnight. Antibodies against mouse or human CCR2, CCR5, CXCR3, CXCR4, GAPDH, IDO, PKC-α, PKC-δ, phospho-STAT1, and STAT1 were purchased from Cell Signaling Technology. After washing, membranes were incubated with horseradish peroxidase- conjugated secondary antibody and signals were detected by enhanced chemiluminescence (Amersham Pharmacia Biotech, Piscataway, NJ, USA) 33, 42.

### Phenotyping

Cells were stained for 15 min at 4 °C with FITC-conjugated antibody against mouse CD4 or CD138 (BD Biosciences). Alternatively, cells were fixed using a Cytofix-Cytoperm Kit (BD Biosciences) and then stained with anti-Foxp3-APC or anti-IgG-APC antibody (eBiosciences, San Diego, CA, USA). Cells were analyzed using a flow cytometer (FACSCalibur) and the data were processed using Cell Quest Pro Software 44.

### Statistical analysis

Data are presented as the mean ± SEM of at least three independent *in vitro* experiments performed in triplicate or six mice. To determine statistical significance, *p*-values were calculated using one-way ANOVA (GraphPad Software, San Diego, CA, USA).

## Results

### mMSCs inhibit mB cells in a CXCL12-dependent manner

**Figure 1:**
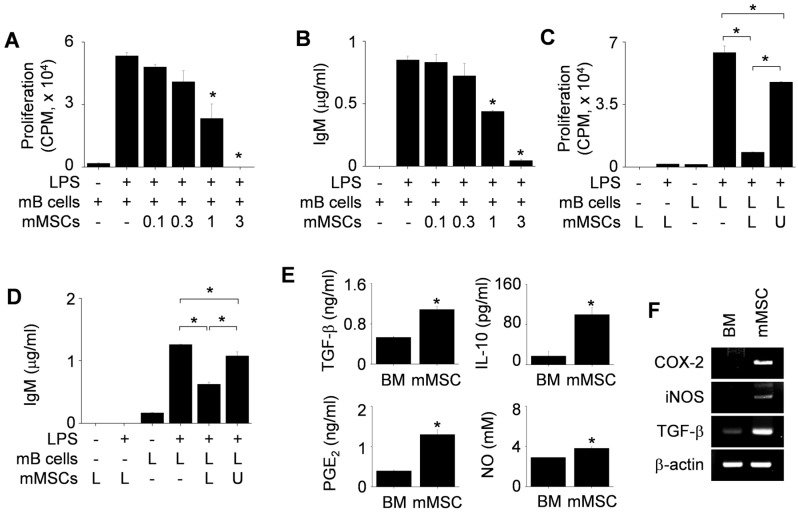
** Effects of mMSCs on the proliferation of and IgM production by mB cells. (A-B)** mMSCs (0.1-3 × 10^4^ cells/well) and MRL*.Fas*^lpr^ mB cells (1 × 10^5^ cells/well) were co-cultured for 72 h. LPS (1 μg/mL) was used to activate mB cells. The proliferation of and IgM production by mB cells were measured by the mitogen assay (A) and ELISA (B), respectively. **(C-D)** mMSCs (1 × 10^4^ cells/well) were added to the upper (U) or lower (L) wells of transwell plates and mB cells (1 × 10^5^ cells/well) to the L wells. After incubation with LPS for 72 h, the mitogen assay (C) and ELISA (D) were performed. **(E)** The levels of TGF-β, IL-10, and PGE_2_ accumulated in the culture medium of BM cells and mMSCs for 24 h were measured by ELISA. NO level was measured with Griess reagent. **(F)** Expression levels of COX-2, iNOS, and TGF-β mRNAs in BM cells and mMSCs were assessed by RT-PCR. ^*^p < 0.01 (n = 3).

First, we used mMSCs generated from BM cells of C3H/HeN mice (H-2^k^) and splenic B cells isolated from lupus-prone MRL*.Fas*^lpr^ mice (H-2^k^). We found that mMSCs inhibited the proliferation of (Figure 1A) and IgM production (Figure 1B) by LPS-treated mB cells in a dose-dependent manner. mMSCs also inhibited the IgG production by mB cells activated with anti-CD40 antibody, IL-4, and IL-21 (Figure S1A). To assess whether mMSCs inhibit mB cells in a soluble factor- or contact-dependent manner, we used a transwell assay. When we added mMSCs and mB cells to the lower wells, thereby allowing cell-cell contact, mMSCs strongly inhibited mB cell proliferation (Figure 1C) and IgM production (Figure 1D). When we added mMSCs to the upper wells and mB cells to the lower wells, thereby preventing direct cell-cell contact, mMSCs inhibited mB cell functions much more weakly (Figure 1C-D). These data imply that mMSCs inhibit mB cell functions mainly in a contact-dependent manner and partially in a soluble factor-dependent manner. We confirmed that mMSCs produced the immunosuppressive soluble factors TGF-β, IL-10, PGE_2_, and NO (Figure 1E-F).

**Figure 2:**
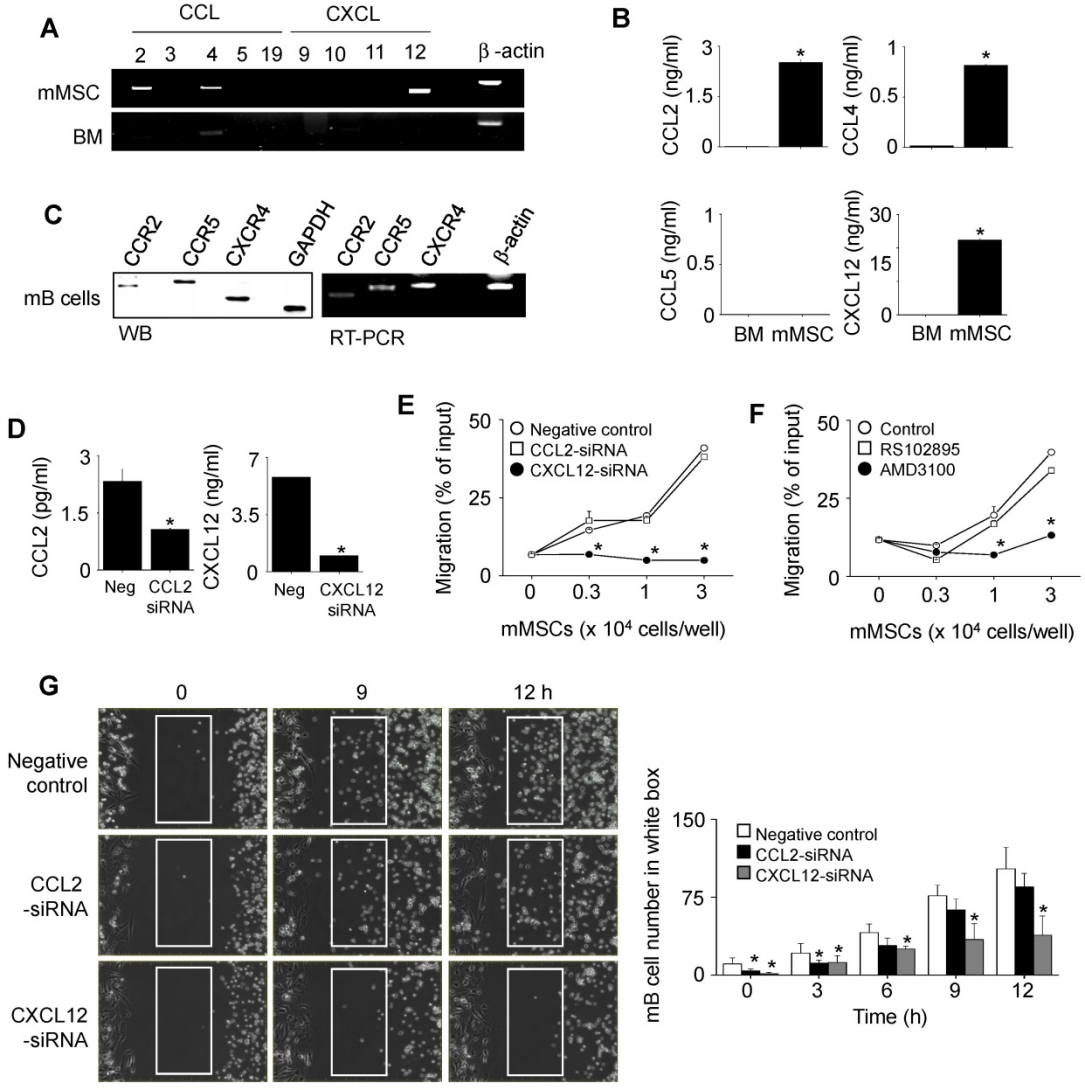
** Effects of mMSCs on the migration of mB cells. (A)** Expression levels of chemokine mRNAs in BM cells and mMSCs were assessed by RT-PCR.** (B)** The levels of CCL2, CCL4, CCL5, and CXCL12 accumulated in culture medium of BM cells and mMSCs for 24 h were measured by ELISA. **(C)** Expression levels of chemokine receptors and their mRNAs in mB cells were assessed by western blotting and RT-PCR, respectively. **(D)** mMSCs were transfected with negative (neg) control, CCL2, or CXCL12 siRNA for 48 h. The levels of CCL2 and CXCL12 accumulated in culture medium for 24 h were measured by ELISA.** (E-F)** CMFDA-labeled mB cells (1 × 10^5^ cells/well) were added to the upper wells of transwell plates with a 5-μm insert. mMSCs (0.3-3 × 10^4^ cells/well), which had been transfected with negative control, CCL2-siRNA, or CXCL12-siRNA, were added to the lower wells (E). CMFDA-labeled mB cells were pre-treated with dimethyl sulfoxide (0.1%, Control), CCR2 antagonist RS102895 (30 μg/mL), or CXCR4 antagonist AMD3100 (300 μg/mL) for 1 h, washed three times, and added to the upper wells. mMSCs (0.3-3 × 10^4^ cells/well) were added to the lower wells (F). After 1.5 h, the number of CMFDA-labeled mB cells migrating to the lower well was determined. **(G)** For time-lapse imaging, mMSCs (70 μL of 0.3 × 10^6^ cells/mL) were seeded into the left chamber and mB cells (70 μL of 1 × 10^6^ cells/mL) into the right chamber of culture-insert μ-Dish^35mm^ culture dishes. Images were acquired every 2 min for 12 h after 1-h pre-incubation. Representative photos are shown (n = 3). The numbers of mB cells passing through the white boxes are shown. ^*^p < 0.01 (n = 3).

Next, we examined how mMSCs inhibited mB cell functions in a contact-dependent manner. To examine the role of chemokines, we assessed the expression profiles of chemokines in mMSCs and chemokine receptors in mB cells. mMSCs expressed mRNAs for CCL2, CCL4, and CXCL12 (Figure 2A), and the corresponding proteins were detectable by ELISA in conditioned medium (Figure 2B). We also confirmed that mB cells expressed mRNAs and proteins of CCR2, CCR5, and CXCR4 (Figure 2C). Then we used siRNAs to knock down CCL2 and CXCL12 in mMSCs (Figure 2D) and performed transwell assay to assess mB cell migration towards mMSCs at the population level. mB cells migrated well to mMSCs transfected with negative control or CCL2 siRNAs, but not to mMSCs transfected with CXCL12 siRNA (Figure 2E). mB cells treated with the CXCR4 antagonist AMD3100 showed little migration toward mMSCs, while mB cells treated with the CCR2 antagonist RS102895 migrated well to mMSCs (Figure 2F). To confirm these results, we used time-lapse imaging at the single-cell level. We placed mMSCs transfected with negative control siRNA, CCL2 siRNA, or CXCL12 on the left side of an imaging chamber and mB cells on the right side and acquired images every 2 min for 12 h (Movie S1). Representative images collected at 2-h intervals are shown in Figure 2G. The number of mB cells passing through the white box at each time point showed that negative control- and CCL2 siRNA-transfected mMSCs induced mB cell migration towards them, whereas CXCL12 siRNA-transfected mMSCs did not (Figure 2G). Overall, these data suggest that CXCL12 produced by mMSCs induces mB cell migration.

### PMA-primed hMSCs inhibit hB cells in a CXCL10-dependent manner

**Figure 3:**
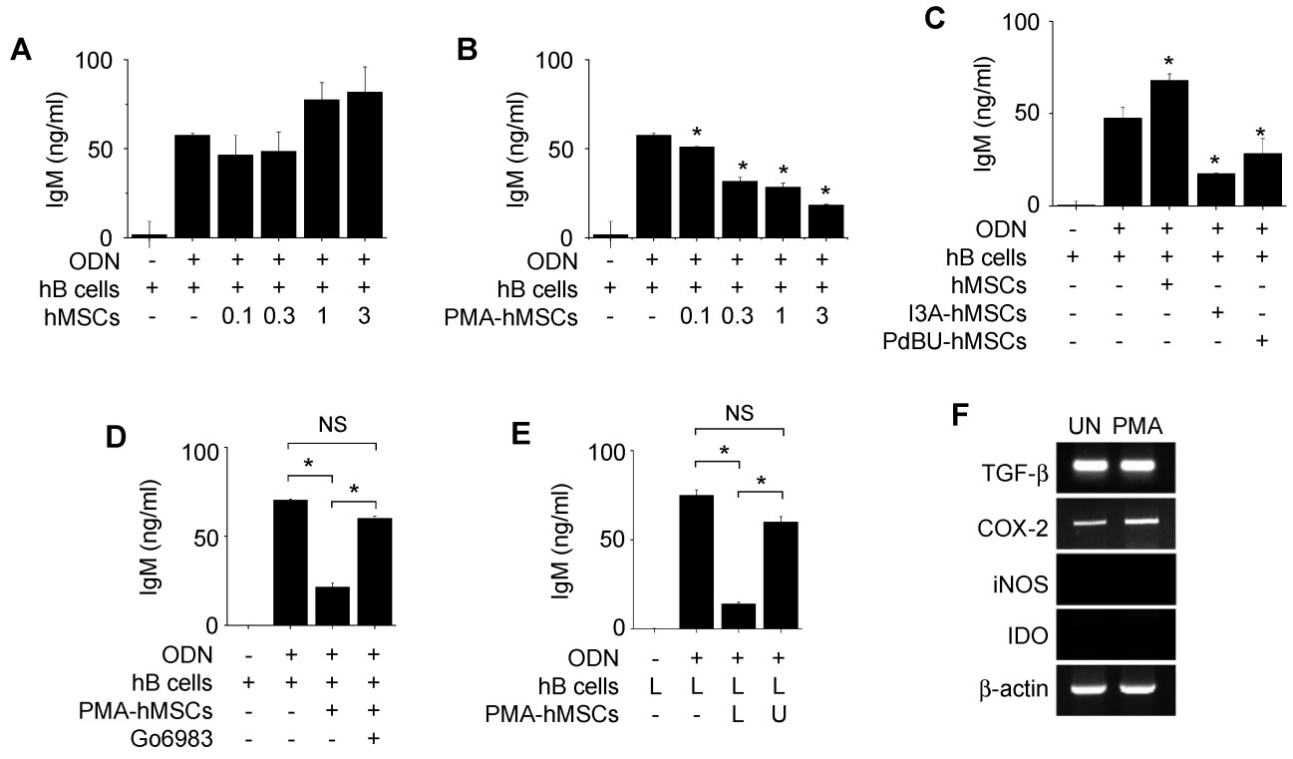
** Effects of PMA-hMSCs on IgM production by hB cells. (A)** hMSCs (0.1-3 × 10^3^ cells/well) and hB cells (1 × 10^5^ cells/well) were co-cultured for 72 h. **(B)** hMSCs were treated with PMA (10 ng/mL) for 24 h and washed three times with medium. PMA-treated hMSCs (PMA-hMSCs; 0.1-3 × 10^3^ cells/well) and hB cells (1 × 10^5^ cells/well) were co-cultured for 72 h. **(C)** hMSCs were treated with ingenol-3-acetate (I3A, 10 µg/mL) or phorbol 12,13-dibutyrate (PdBU, 10 µg/mL) for 24 h and washed three times with medium. Chemical-treated hMSCs (1 × 10^3^ cells/well) were co-cultured with hB cells (1 × 10^5^ cells/well) for 72 h. **(D)** hMSCs were treated with PMA in the presence or absence of the PKC inhibitor Go6983 (1 μg/mL) for 24 h. PMA-hMSCs were washed three times with medium. PMA-hMSCs (1 × 10^3^ cells/well) were co-cultured with hB cells (1 × 10^5^ cells/well) for 72 h. **(E)** PMA-hMSCs (1 × 10^3^ cells/well) were added to the lower wells and hB cells (1 × 10^5^ cells/well) to the upper wells of transwell plates with a 5-μm insert. CpG-oligodeoxynucleotide (ODN, 5 μg/mL) was used to activate hB cells. IgM production by hB cells was measured by ELISA (A-E). **(F)** Total RNA was isolated from chemically untreated hMSCs (UN) or PMA-treated hMSCs (PMA). Gene expression levels were assessed by RT-PCR. ^*^p < 0.01 (n = 3).

Next, we examined the effects of hMSCs generated from human BM cells on hB cells isolated from peripheral blood mononuclear cells of healthy donors. Unexpectedly, hMSCs did not inhibit IgM production by hB cells (Figure 3A). Thus, we screened a number of chemicals for their ability to activate hMSCs to inhibit hB cells (Table S1). Among these chemicals, three protein kinase C (PKC) activators (PMA, phorbol 12,13-dibutyrate, and ingenol 3-angelate) activated hMSCs so that they inhibited IgM production by ODN-treated hB cells (Figure 3B-C). PMA-primed hMSCs (called PMA-hMSCs hereafter) inhibited IgG production by hB cells activated with anti-CD40 antibody, IL-4, and IL-21 (Figure S1B). PKC inhibitor Go6983 abolished the ability of PMA-hMSCs to inhibit hB cells (Figure 3D). When we added PMA-hMSCs and hB cells together to the lower wells of transwell plates, PMA-hMSCs strongly inhibited hB cell IgM production, but, when we separated them, PMA-hMSCs did not (Figure 3E), suggesting that PMA-hMSCs inhibited hB cells mainly in a contact-dependent but not soluble factor-dependent manner. PMA did not increase the expression levels of immunosuppressive soluble factors, such as TGF-β, COX2, iNOS, or IDO, in hMSCs compared with naïve hMSCs (Figure 3F). PMA did not affect the phenotypes (Figure S2A), viability (Figure S2B), and proliferation (Figure S2C) of hMSCs. In addition, PMA did not affect the gene expression of IL-6 and IL-10 by hMSCs (Figure S2D).

**Figure 4:**
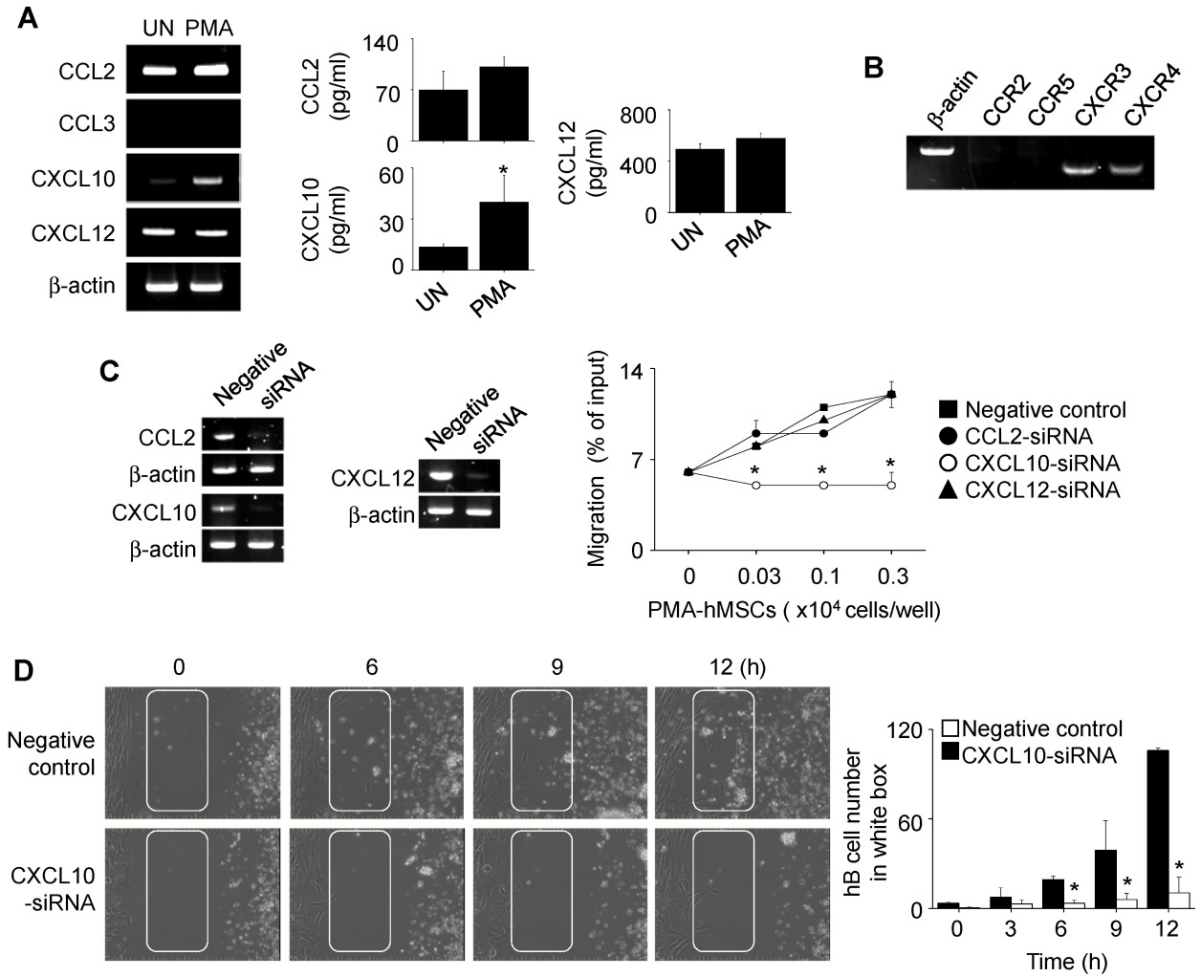
** Effects of PMA on the migration of hMSCs. (A)** hMSCs were activated with PMA for 24 h. Chemokine expression levels were measured by RT-PCR and ELISA. UN, untreated. **(B)** Expression levels of chemokine receptors in hB cells were assessed by RT-PCR. **(C)** PMA-hMSCs were transfected with negative-control, CCL2, CXCL10, or CXCL12 siRNA for 48 h, and chemokine expression levels were analyzed by RT-PCR. PMA-hMSCs (0.03-0.3 × 10^4^ cells/well) were added to the lower wells and CMFDA-labeled hB cells (1 × 10^5^ cells/well) to the upper wells of transwell plates with a 5-μm insert. After 1.5 h, the number of CMFDA-labeled hB cells migrating to the lower well was determined. **(D)** For time-lapse imaging, hMSCs (70 μL of 0.3 × 10^6^ cells/mL) were seeded into the left chamber and hB cells (70 μL of 1 × 10^6^ cells/mL) into the right chamber of culture-insert μ-Dish^35mm^ culture dishes. Images were acquired every 2 min for 12 h. Representative photos are shown. The numbers of hB cells passing through the white boxes are shown. ^*^p < 0.01 (n = 3).

Next, we investigated how PMA-hMSCs inhibited hB cell functions in a contact-dependent manner. PMA increased the expression of CXCL10, but not of CCL2, CCL3, or CXCL12, by hMSCs (Figure 4A). hB cells expressed the CXCL10 receptor CXCR3 (Figure 4B). We decreased the expression of CCL2, CXCL10, and CXCL12 in PMA-hMSCs with specific siRNAs and used them in transwell assay. PMA-hMSCs transfected with CXCL10 siRNA did not induce hB cell migration, but other PMA-hMSCs attracted hB cells (Figure 4C). These results were confirmed by time-lapse imaging (Movie S2). Representative images collected at 2-h intervals are shown in Figure 4D. The number of hB cells passing through the white box at each time point showed that CXCL10 siRNA-transfected PMA-hMSCs did not induce hB cell migration (Figure 4D). Overall, these data suggest that PMA-hMSCs use CXCL10 to attract hB cells, unlike mMSCs, which use CXCL12.

### PMA-hMSCs inhibit hB cells in a PD-L1-dependent manner

**Figure 5:**
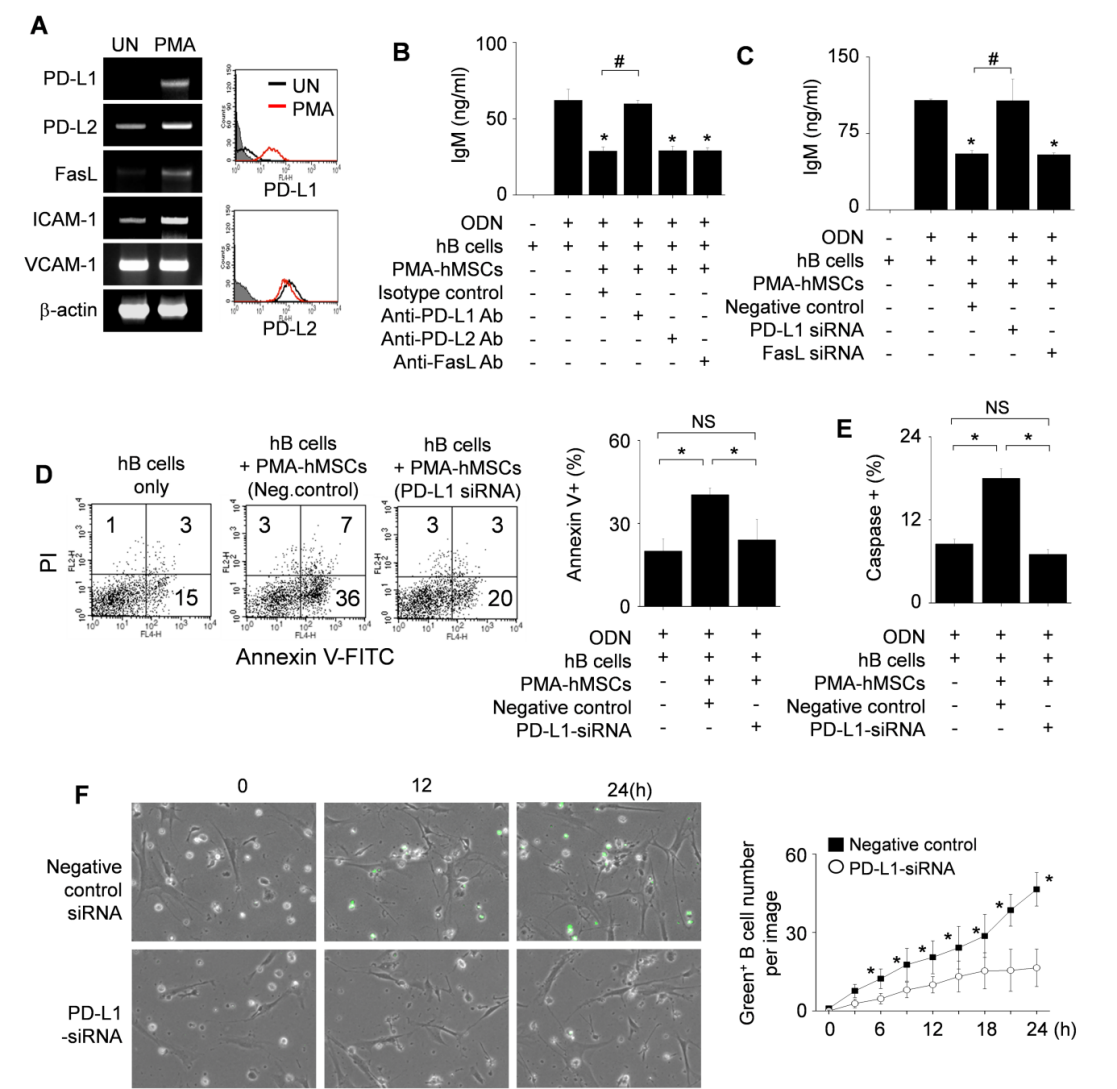
** Effects of PMA-hMSCs on the viability of hB cells. (A)** hMSCs were activated with PMA for 24 h. Expression levels of the death ligands PD-L1, PD-L2, and FasL were measured by RT-PCR and flow cytometric analysis. UN, untreated. **(B-C)** PMA-hMSCs (1 × 10^3^ cells/well) were co-cultured with hB cells (1 × 10^5^ cells/well) for 72 h in the presence of blocking antibodies against PD-L1, PD-L2, or FasL (B). PMA-hMSCs, which had been transfected with PD-L1 or FasL siRNA, were co-cultured with hB cells (C). ODN (5 μg/mL) was used to activate hB cells. IgM production by hB cells was measured by ELISA assay. **(D-F)** PMA-hMSCs (0.1 × 10^6^ cells), which had been transfected with PD-L1 siRNA, were cultured with hB cells (1 × 10^6^ cells) in 35-mm culture dishes for 24 h. hB cells were stained with anti-CD19-APC and then stained with FITC-Annexin V and propidium iodide (PI). Cells were analyzed using a flow cytometer (D). hB cells were stained with anti-CD19-APC and Intracellular Caspase Detection ApoStat kit and analyzed using a flow cytometer (E). CellEvent Caspase-3/7 Green ReadyProbes Reagent was added to the culture of hB cells and hMSCs, and the cells were imaged every 10 min for 24 h with a Biostation IM-Q microscope (Nikon). Green fluorescent cells were considered apoptotic (F). ^*^p < 0.01 (n = 3).

Next, we examined how PMA-hMSCs directly inhibit hB cell functions after contact. PMA strongly increased the expression of PD-L1 and weakly that of PD-L2 and FasL by hMSCs (Figure 5A). Anti-PD-L1 blocking antibody abolished the inhibitory capacity of PMA-hMSCs, but anti-PD-L2 and anti-FasL antibodies did not (Figure 5B). PMA-hMSCs transfected with PD-L1 siRNA did not inhibit B cells, but PMA-hMSCs transfected with FasL siRNA inhibited hB cells well (Figure 5C).

Then, we examined whether PD-L1-expressing PMA-hMSCs can induce apoptosis of hB cells in three ways. First, we stained hB cells with annexin V and propidium iodide. PMA-hMSCs markedly increased the proportion of annexin V-positive apoptotic hB cells, but PMA-hMSCs transfected with PD-L1 siRNA did so only weakly (Figure 5D). Second, we labeled hB cells with FITC-VAD-FMK (ApoStat), a cell-permeable fluorescent pan-caspase probe. PMA-hMSCs increased pan-caspase activity in hB cells, but PMA-hMSCs transfected with PD-L1 siRNA did not (Figure 5E). Third, we labeled hB cells with the DEVD peptide conjugated to a nucleic acid-binding dye (CellEvent Caspase-3/7 Green ReadyProbes Reagent). Upon activation of caspase-3/7 in apoptotic cells, DEVD is cleaved and the free dye binds DNA, generating bright green fluorescence. By using time-lapse imaging, we showed that caspase-3/7 activity was higher in PMA-hMSCs than in PMA-hMSCs transfected with PD-L1 siRNA (Movie S3 and Figure 5F). Overall, these data suggest that PMA-hMSCs activates caspases in a PD-L1-dependent manner, followed by hB cell apoptosis.

### PMA-hMSCs are more efficient than naïve hMSCs in ameliorating lupus progression in MRL*.Fas*^lpr^ mice

**Figure 6:**
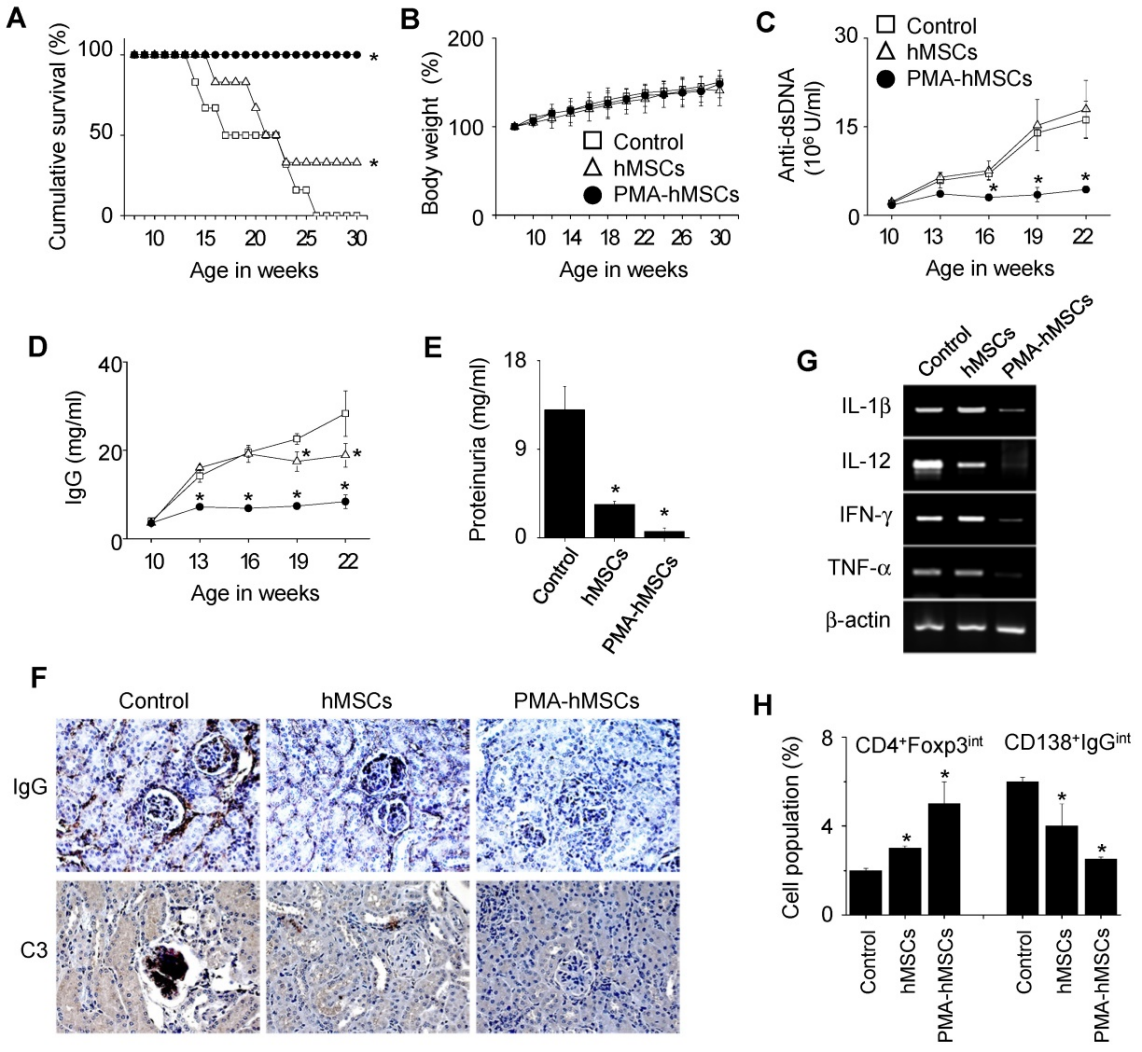
***In vivo* efficacy of PMA-hMSCs in MRL.*Fas*^lpr^ mice. (A-B)** MRL*.Fas*^lpr^ mice were intravenously injected with PBS (control), naïve hMSCs (4 × 10^4^ cells /injection), or PMA-hMSCs (4 × 10^4^ cells/injection) once at the age of 12 weeks. Survival was measured every week (A) and body weight (B) every 2 weeks up to 30 weeks of age. ^*^p < 0.01 (n = 6).** (C-H)** Injections were performed as in (A). Surviving mice were sacrificed at the age of 22 weeks. The serum levels of anti-dsDNA IgG (C) and total IgG (D) were measured every 3 weeks. Proteinurea levels were measured at the age of 22 weeks (E). Kidney sections were stained with antibodies against IgG and C3 complement (F). Total RNA was isolated from spleen cells and the expression levels of inflammatory cytokine genes (IL-1β, IL-12, IFN-γ, and TNF-α) were examined by RT-PCR (G). The ratios of Foxp3-expressing CD4^+^ Treg cells (CD4^+^Foxp3^int^) and IgG-producing CD138^+^ plasma cells (CD138^+^IgG^int^) in the spleen were measured by flow cytometry (H). ^*^p < 0.01 (n = 6).

**Figure 7:**
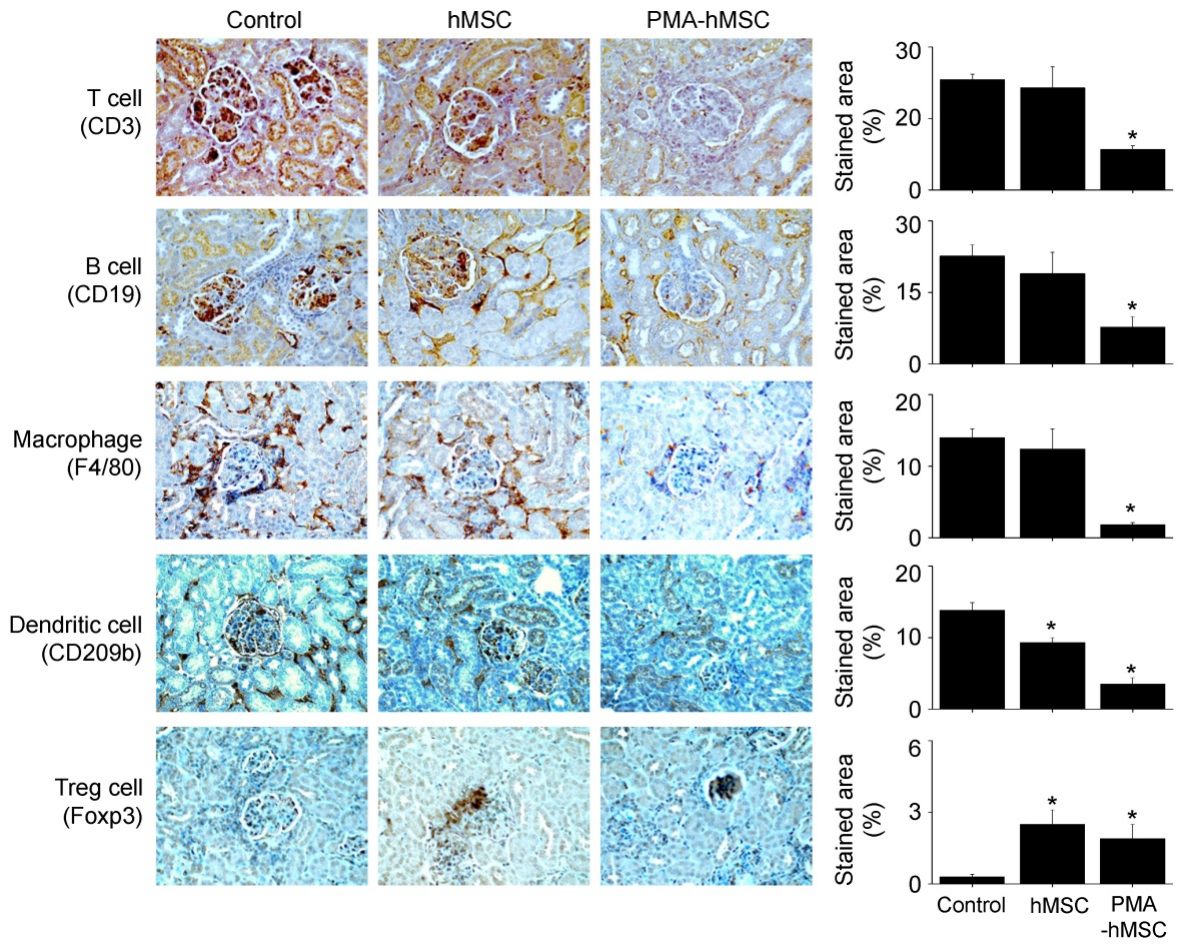
** Immunohistochemical analysis.** Kidney sections used in Figure 6F were stained with antibodies against CD3 (T cells), CD19 (B cells), F4/80 (macrophages), CD209b (dendritic cells), or Foxp3 (Treg cells).

Finally, we examined the therapeutic activity of PMA-hMSCs in lupus-prone MRL*.Fas*^lpr^ mice. Our preliminary experiments showed that hMSCs at 4 × 10^6^ cells/injection completely prevented lupus progression in MRL*.Fas*^lpr^ mice. To compare the efficacy of hMSCs and PMA-hMSCs, we therefore injected lower numbers of hMSCs and PMA-hMSCs (4 × 10^4^ cells/injection). All mice (n = 6) that received PMA-hMSCs survived up to 30 weeks of age, which was much longer than control and hMSC-injected groups (Figure 6A). Regardless of PMA priming, hMSCs did not affect body weight (Figure 6B), and no untoward effects were noted. In another experiment, when 50% of control mice survived (22 weeks of age), we sacrificed the surviving mice. The serum levels of anti-dsDNA (Figure 6C) and total IgG (Figure 6D) antibodies and the levels of protein in urine (Figure 6E) were much lower in PMA-hMSC-treated mice than in the control and hMSC-treated mice. The deposition of IgG and C3 complement in the kidney was significantly decreased in PMA-hMSC-treated mice in comparison with the control and hMSC-treated mice (Figure 6F). The expression of all inflammatory cytokines tested (IL-1β, IL-12, IFN-γ, and TNF-α) in the spleen was also lower in PMA-hMSC-treated mice than in the control and hMSC-treated mice (Figure 6G). The frequency of Foxp3-expressing CD4^+^ Treg cells was higher and that of IgG-producing CD138^+^ plasma cells was lower in the spleens of PMA-hMSC-treated mice than in those of the control and hMSC-treated mice (Figure 6H). The infiltration of T cells, B cells, macrophages, and dendritic cells into the kidney was significantly decreased in PMA-hMSC-treated mice in comparison with the control and hMSC-treated mice (Figure 7). In contrast, the infiltration of Treg cells into the kidney was significantly increased in PMA-hMSC-treated mice in comparison with the control mice (Figure 7). Overall, our data suggest that PMA-hMSCs ameliorate the development of SLE-like disease in MRL*.Fas*^lpr^ mice more effectively than do naïve hMSCs.

**Figure 8:**
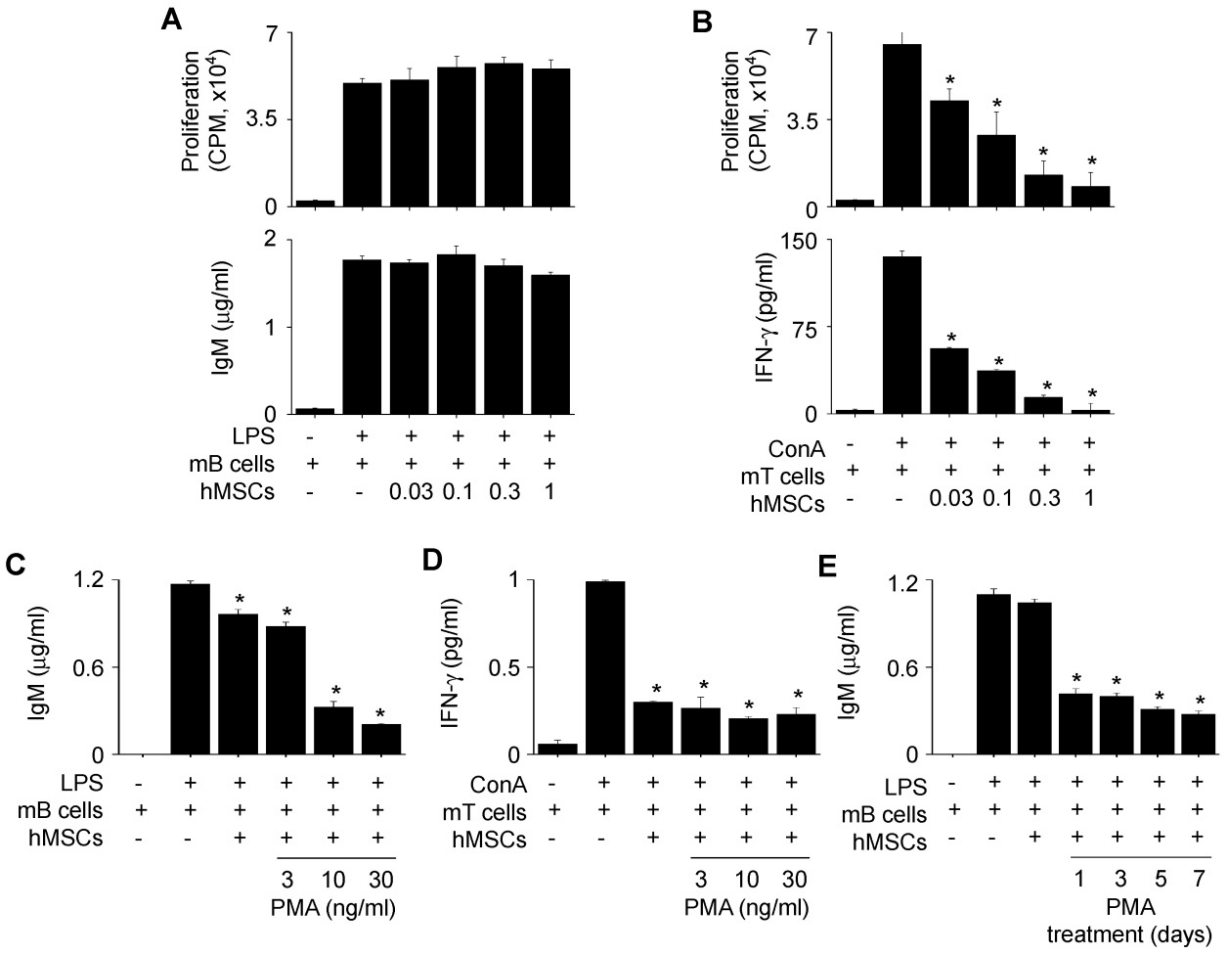
** Effects of PMA-hMSCs on xenogeneic mB cells. (A)** hMSCs (0.03-1 × 10^4^ cells/well) and MRL*.Fas*^lpr^ mB cells (1 × 10^5^ cells/well) were co-cultured for 72 h. LPS (1 μg/mL) was used to activate mB cells. The proliferation of and IgM production by mB cells were measured by the mitogen assay and ELISA, respectively. **(B)** hMSCs (0.03-1 × 10^4^ cells/well) and MRL*.Fas*^lpr^ mT cells (1 × 10^5^ cells/well) were co-cultured for 72 h. Concanavalin A (ConA, 1 μg/mL) was used to activate mT cells. The proliferation of and IFN-γ production by mT cells were measured by the mitogen assay and ELISA, respectively. **(C-D)** hMSCs (1 × 10^4^ cells/well) were activated with PMA (3-30 ng/mL) for 24 h and then co-cultured with mB cells (1 × 10^5^ cells/well) (C) or mT cells (1 × 10^5^ cells/well) (D). **(E)** hMSCs were activated with PMA (10 ng/mL) for 1 to 7 days and then co-cultured with mB cells. ^*^p < 0.01 (n = 3).

Since we injected PMA-hMSCs into the xenogeneic MRL*.Fas*^lpr^ mice, we examined whether PMA-hMSCs inhibited mB cells. hMSCs did not inhibit the proliferation of or IgM production by mB cells from MRL*.Fas*^lpr^ mice (Figure 8A). However, hMSCs inhibited well the proliferation of and IFN-γ production by concanavalin A (ConA)-activated T cells from the same mice (Figure 8B). PMA-hMSCs inhibited both IgM production by mB cells (Figure 8C) and IFN-γ production by mouse T cells (Figure 8D). We found that 1-day priming of hMSCs with PMA was enough to activate hMSCs to inhibit mB cells (Figure 8E). We also proved that mMSCs, hMSCs, and PMA-hMSCs inhibited IFN-γ production by T cells activated with anti-CD3 and anti-CD28 antibody, which mimicked TCR-dependent T cell activation (Figure S1C-D). Overall, our data suggest that the improved efficacy of PMA-hMSCs in MRL*.Fas*^lpr^ mice might be due to the dual inhibition of T and B cells.

## Discussion

Two approaches have been proposed to improve the functions of MSCs. One is MSC priming with cytokines and growth factors. IFN-γ, TNF-α, IL-17, and IL-1β are well known to enhance the immunosuppressive properties of MSCs by upregulating the secretion of IDO, PGE_2_, and TGF-β 45-49. The second approach is MSC priming with chemicals, which might be more cost effective than priming with cytokines. Valproic acid, sphingosine-1-phosphate, 5-aza-2'-deoxycytidine, dimethyloxalylglycine, 2-chloro-N6-cyclopentyl-adenosine, rapamycin, and all-*trans* retinoic acid enhance the differentiation, proliferation, and tissue-homing ability of MSCs 50-55. In this study, we investigated in detail the effects of chemicals on the ability of MSCs to suppress the functions of B cells and found that PMA improves the suppressive activity of hMSCs on hB cells. Compared to naïve hMSCs, PMA-hMSCs strongly inhibited IgM production by hB cells.

The first question addressed in this study is how PMA-hMSCs inhibit B cells. It is well documented that MSCs can inhibit T cells through both contact- and soluble factor-dependent mechanisms 16-18, 24, 56, 57. However, PMA-hMSCs inhibit hB cells mainly in a contact-dependent manner, but not through soluble factors. When those cells were separated in our transwell assay, PMA-hMSCs did not affect hB cells, but allowing their contact resulted in PMA-hMSCs strongly and significantly inhibiting hB cells.

The second question is how PMA-hMSCs contact hB cells. Our data suggest an important role of CXCL10 produced by adherent PMA-hMSCs in migration of non-adherent hB cells. It is well known that serum levels of CXCL10 are increased in SLE patients and are strongly correlated with disease activity 58. In lupus-prone MRL/lpr mice, the number of CXCR3-positive plasma B cells is increased in the secondary lymphoid organs during the development of lupus nephritis 59. Importantly, the nephritic kidney shows high expression of CXCL10 60, which increases the migration of CXCR3-positive plasma B cells from the secondary lymphoid organs into the inflamed kidney. Our data that PMA-hMSCs transfected with CXCL10 siRNA did not attract B cells suggest an additional role of CXCL10 in that this chemokine optimizes the interaction between MSCs and B cells, which might happen in the nephritic kidney. CCL2 and CXCL12 appear not to be involved in PMA-hMSC-induced attraction of hB cells. Two interesting aspects are worth noting. First, mMSCs use a different chemokine, CXCL12, to induce mB cell migration. CXCL12 siRNA-transfected mMSCs were unable to induce mB cell migration, and a CXCR4 antagonist blocked mB cell migration to mMSCs. Second, MSCs use CCL2 to attract T cells. CCL2-deficient MSCs can neither attract T cells nor inhibit T cell functions 4. MSCs use CCL2 for the recruitment and subsequent contact-dependent inhibition of inflammatory Th17 cells and for the homing of Tregs and myeloid-derived suppressor cells to the damaged organs in models of experimental autoimmune encephalomyelitis and experimental autoimmune uveitis 61, 62. Overall, our data suggest that MSCs use different chemokines to attract T and B cells in a species-dependent manner.

The next question is how PMA-hMSCs inhibit hB cells after contact. Our data suggest a key role for PD-L1. PD-1 is broadly expressed on B cells and other immune cells, including T cells 63. Although PD-1 interacts with PD-L1 and PD-L2, PD-L1 might act as the primary ligand of PD-1 64. Unlike PD-L2, PD-L1 induces a considerable conformational change of PD-1 and is widely expressed on most cell types, including dendritic cells, macrophages, endothelial cells, and placenta cells 64, 65. PD-L1 has been extensively studied as a negative regulator of the immune response that enables tumor cells to escape T cell immunity 66, 67, and as an inducer of peripheral tolerance to prevent autoimmune diseases 66, 68. MSCs highly express PD-L1 upon activation with IFN-γ or TNF-α, although naïve MSCs express it very weakly 64, 69, 70. IFN-γ treated MSCs inhibit cytokine production, proliferation, and differentiation of T cells and induce T cell apoptosis in a PD-L1-dependent manner, although some discrepancies are reported depending on the experimental conditions 63, 64, 69, 71-73. MSCs inhibit B cell antibody production in a PD-1-dependent contact-inhibition manner 69. In agreement with these data, our data show that PMA-hMSCs inhibit hB cell functions in a PD-L1-dependent manner, which was proved by siRNA and blocking-antibody experiments.

The implications of our study are limited by several caveats. First, we did not clarify the signaling in PMA-hMSCs. PMA activates PKCs and subsequently activates several transcription factors, such as NF-AT and NF-κB 74, 75, which are able to increase the mRNA expression of CXCL10 and PD-L1 66, 76, 77. It is also unclear how long PKC activation in hMSCs is maintained after washing out PMA. Our preliminary experiments demonstrated that the amount of PKCs in the cytosol of hMSCs decreased from 1 h and PKC almost disappeared 24 h after the onset of PMA treatment (Figure S3A). After removing PMA, the amount of PKCs in the cytosol of hMSCs was maintained at low level up to 3 days and increased from 4 days (Figure S3B). The gene expression of PD-L1 in hMSCs was maintained up to 2 days and decreased from 3 days after washing out PMA (Figure S3C). Further studies to delineate the detailed signaling cascades in PMA-hMSCs are needed, because elucidation of the underlying mechanism would enable us to develop a strategy for the generation of MSCs with potent immunosuppressive activities. Second, we did not study the negative role of PD-1 signaling in B cell activation. Although much has been learned about the inhibitory mechanisms of PD-1 signaling in T cell activation, little is known about how PD-1 inhibits B cell activation 71. Thus, further studies are required to reveal the detailed inhibitory mechanisms of PD-1 signaling in B cell activation. Third, our experimental system, which uses MSCs and purified B cells, is not identical to *in vivo* conditions, since the interaction between MSCs and B cells is extremely complicated and is likely affected by many different factors *in vivo*. However, our system might be optimal for investigating their interaction because it is easily controllable and can be expected to yield reproducible data since there are few interfering factors 78.

Despite these shortcomings, the results of this study provide several insights into the mechanisms of B cell inhibition by MSCs. First, mMSCs inhibit mB cells, but hMSCs do not inhibit hB cells, which suggests a species difference in MSC effects on B cells. Second, phorbol ester priming of hMSCs can increase their immunosuppressive capacity against hB cells. Third, PMA-hMSCs inhibit hB cells via unique mechanisms using CXCL10 and PD-L1. Fourth, in comparison with naïve hMSCs, PMA-hMSCs have better therapeutic efficacy *in vivo* in an SLE mouse model. Most importantly, our data provide a clue on how to generate hMSCs effective for the treatment of patients with SLE and other autoimmune diseases characterized by excessive B cell activation.

## Supplementary Material

Supplementary figures and tables.

Supplementary movie S1.

Supplementary movie S2.

Supplementary movie S3.

## Abbreviations

BM: bone marrow
ConA: concanavalin A
IDO: indoleamine 2,3-dioxygenase
I3A: ingenol 3-angelate
LPS: lipopolysaccharide
MSCs: mesenchymal stem cells
NO: nitric oxide
ODN: oligodeoxynucleotide
PdBU: phorbol 12,13-dibutyrate
PGE_2_: prostaglandin E_2_
PI: propidium iodide
PMA: phorbol myristate acetate
siRNAs: small interfering RNAs
SLE: systemic lupus erythematosus
TGF-β: tumor growth factor-beta
