# Supplementary material for: Phorbol ester activates human mesenchymal stem cells to inhibit B cells and ameliorate lupus symptoms in MRL.Faslpr mice
Source: Theranostics. 2020 Aug 13;10(22):10186–99. doi: 10.7150/thno.46835 (PMC7481409; doi:10.7150/thno.46835)
Supplement: Supplementary file 1 — Supplementary figures and tables. [file thnov10p10186s1.pdf]

Supplementary Figures

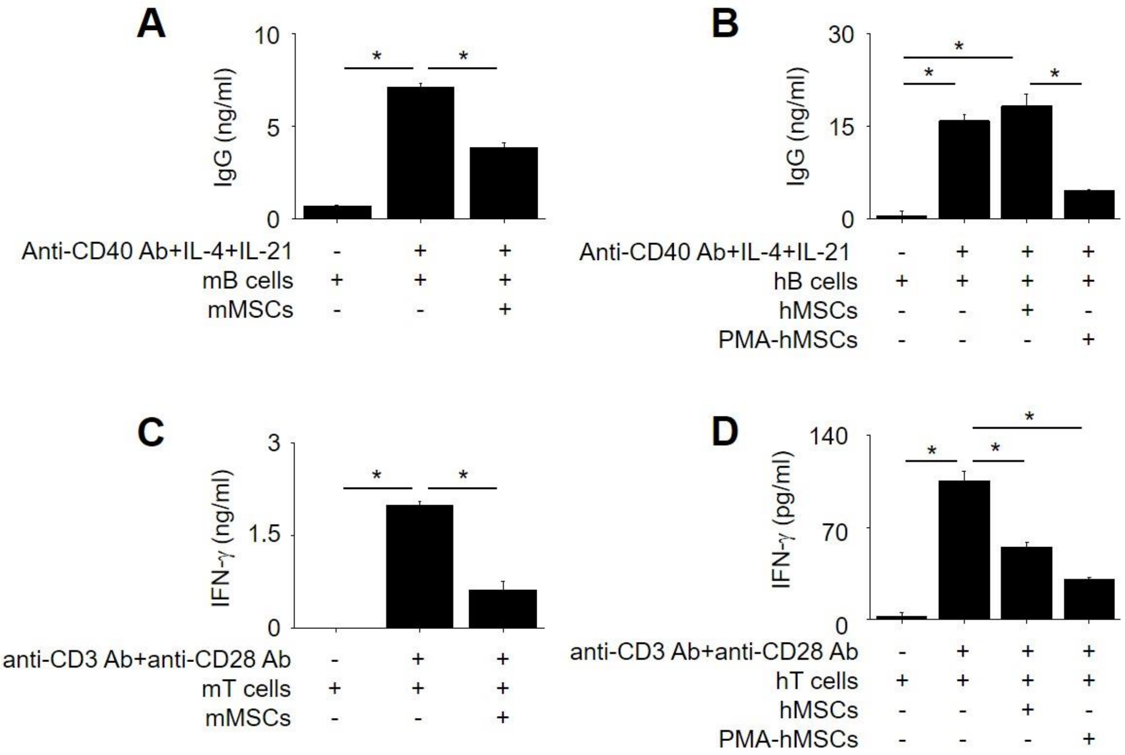

Figure S1. Effects of MSCs on IgG production by B cells and IFN-γ production by T cells. (A–B) mMSCs (1 × 10^4 cells/well) and MRL.*Fas*^{lpr} mB cells (1 × 10^5 cells/well) were cultured for 72 h (A). hMSCs or PMA-hMSCs (1 × 10^3 cells/well) and hB cells (1 × 10^5 cells/well) were cultured for 72 h (B). B cells were activated with anti-CD40 antibody (5 μg/mL), IL-4 (50 ng/mL), and IL-21 (50 ng/mL), and IgG production was measured by ELISA. **(C–D)** mMSCs (1 × 10^4 cells/well) and MRL.*Fas*^{lpr} mT cells (1 × 10^5 cells/well) were cultured for 72 h (C). hMSCs or PMA-hMSCs (1 × 10^3 cells/well) and hT cells (1 × 10^5 cells/well) were cultured for 72 h. T cells were activated with anti-CD3 antibody (2 μg/mL) and anti-CD28 antibody (2 μg/mL), and IFN-γ production was measured by ELISA. *$p < 0.01$ (n = 3).

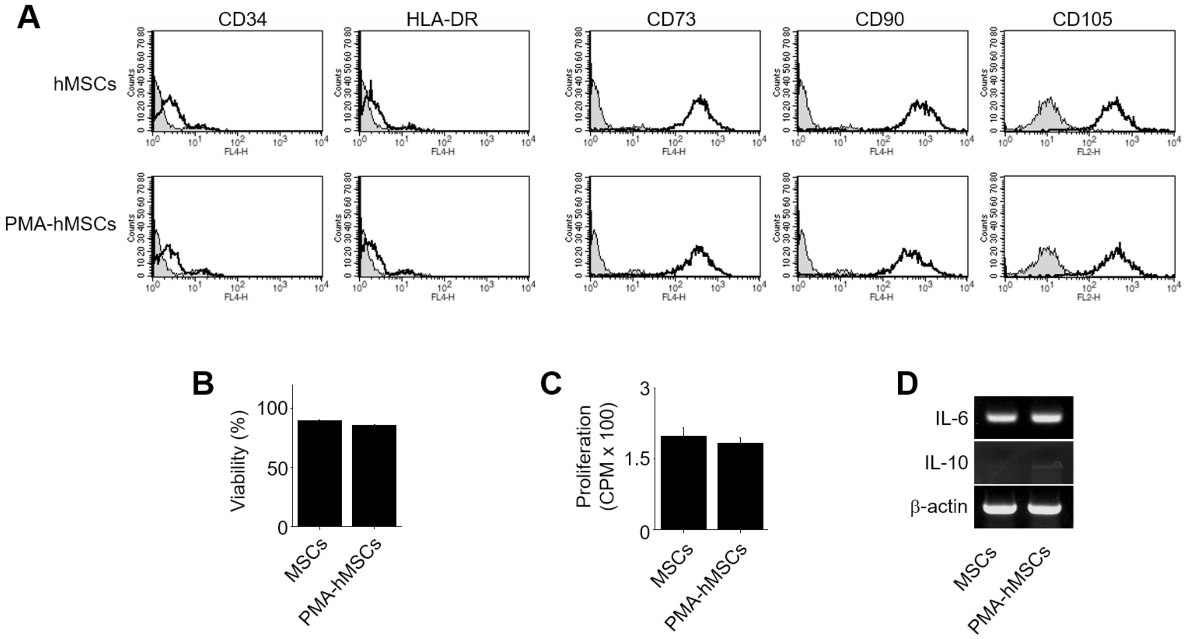

Figure S2. Effects of PMA on phenotypes, viability, and proliferation of hMSCs, and cytokine production by hMSCs. hMSCs were treated with PMA (10 ng/mL) for 24 h and then washed three times with medium. **(A)** Expression of CD34, HLA-DR, CD73, CD90, and CD105 was determined by flow cytometry. **(B)** MSCs were stained with propidium iodide (1 μg/mL) and viability was determined by flow cytometry. **(C)** MSC proliferation was measured by mitogen assay. **(D)** Gene expression of cytokines was assessed by RT-PCR (n = 3).

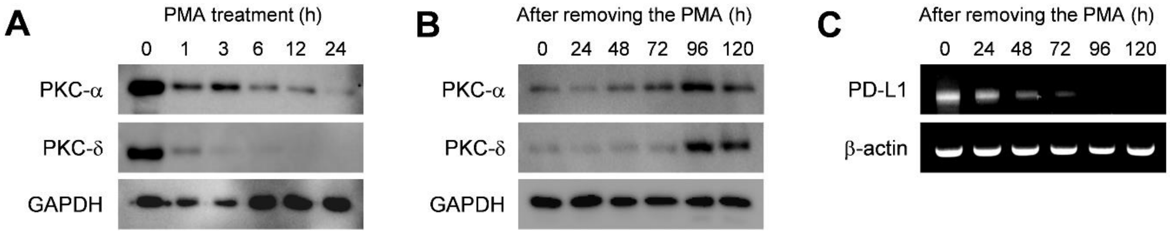

Figure S3. PKC activation in PMA-hMSCs. (A) hMSCs were treated with PMA (10 ng/mL) for 1 to 24 h. Expression levels of PKC-α and -δ were assessed by western blotting. **(B–C)** hMSCs were treated with PMA (10 ng/mL) for 24 h, washed three times with medium, and further cultured with the fresh medium. Expression levels of PKC-α and -δ were assessed by western blotting (B). Expression levels of PD-L1 were assessed by RT-PCR (C).

Supplementary Movies

Movie S1. mB cell migration to mMSCs. mMSCs in the left chamber and mB cells in the right chamber of a culture dish were imaged every 2 min for 12 h. Related to Figure 2G.

Movie S2. hB cell migration to PMA-hMSCs. PMA-hMSCs in the left chamber and hB cells in the right chamber of a culture dish were imaged every 2 min for 12 h. Related to Figure 4D.

Movie S3. Contact between hB cells and PMA-hMSCs. PMA-hMSCs and hB cells were imaged every 5 min for 24 h. Apoptotic hB cells were detected with CellEvent Caspase-3/7 Green ReadyProbes Reagent. Related to Figure 5F.

Supplementary Tables

Table S1. Chemical screening to activate hMSCs to inhibit IgM production by hB cells

Code	Chemical	Concentration	Inhibition (%)[a]
1	Tumor necrosis factor-alpha	100 ng/mL	16
2	Cobalt chloride	500 µM	0
3	Interleukin-1beta	100 ng/mL	0
4	Lipopolysaccharide	1 µg/mL	2
5	Oligodeoxynucleotide	10 µg/mL	3
6	Ascorbic acid	50 µM	17
7	Interferon-gamma	10 ng/mL	83
8	Phorbol 12-myristate 13-acetate	10 ng/mL	55
9	Phorbol 12,13-dibutyrate	1 µg/mL	70
10	Forskolin	100 µg/mL	15
11	Ingenol-3-angelate	10 µg/mL	64
12	Esulone L1	1 µg/mL	0
13	Esulone L2	1 µg/mL	10
14	Kansuinine A	1 µg/mL	15
15	Kansuinine B	1 µg/mL	28
16	Kansuinine D	1 µg/mL	28
17	Kansuinine E	1 µg/mL	2
18	Esulone A	1 µg/mL	2
19	Ingenol	1 µg/mL	2
20	20-Acetylingenol	1 µg/mL	2
21	Kansuiphorin C	1 µg/mL	0
22	3-O-Benzoyl-20-deoxyingenol	1 µg/mL	6
23	5-O-Benzoyl-20-deoxyingenol	1 µg/mL	11
24	3-O-(2′E,4′Z-Decadienoyl)-20-deoxyingenol	1 µg/mL	19
25	3-O-(2′E,4′Z-Decadienoyl)-20-acetylingenol	1 µg/mL	26
26	3-O-(2′E,4′E-Decadienoyl)-20-acetylingenol	1 µg/mL	34
27	5-O-(2′E,4′Z-Decadienoyl)-20-acetylingenol	1 µg/mL	19
28	5-O-(2′E,4′E-Decadienoyl)-20-acetylingenol	1 µg/mL	37
29	Scopoletin	1 µg/mL	0
30	Kansenonol	1 µg/mL	0
31	Fischeliolide A	1 µg/mL	0
32	Fischeliolide B	1 µg/mL	0
33	Fischeliolide C	1 µg/mL	0
34	Fischeliolide D	1 µg/mL	0

35	Ent-11α-Hydroxyabieta-8(14),13(15)-dien-16,12α-olide	1 μg/mL	0
36	11β-Hydroxy-8,14,epoxy-ent-abieta-13(15)-en-16,12α-olide	1 μg/mL	0
37	Jolkinolide B	1 μg/mL	0
38	Yuexiandajisu D	1 μg/mL	0
39	Yuexiandajisu E	1 μg/mL	0
40	Ebractenoid B	1 μg/mL	0
41	Ebractenoid F	1 μg/mL	0
42	Ebractenoid H	1 μg/mL	3
43	Antiquorin	1 μg/mL	12
44	Ebracteolata compound B	1 μg/mL	9
45	6-O-Methylphloroacetophenone	1 μg/mL	3
46	Euphorbia factor L27	1 μg/mL	3
47	Euphorbia factor L28	1 μg/mL	5
48	Epoxyboetirane A	1 μg/mL	12
49	Euphorbia factor L1	1 μg/mL	2
50	Deoxy Euphorbia factor L1	1 μg/mL	0
51	Euphorbia factor L2	1 μg/mL	0
52	Euphorbia factor L3	1 μg/mL	0
53	Euphorbia factor L7a	1 μg/mL	6
54	Euphorbia factor L7b	1 μg/mL	6
55	Euphorbia factor L8	1 μg/mL	14
56	Euphorbia factor L9	1 μg/mL	11
57	Euphorbia factor L17	1 μg/mL	0
58	Euphorbia factor L22	1 μg/mL	0
59	Euphorbia factor L23	1 μg/mL	0
60	Euphorbia factor L24	1 μg/mL	0
61	Euphorbia factor L25	1 μg/mL	0
62	Jolkinol A	1 μg/mL	0
63	Jolkinol-5β,6β-oxide-3-nicotinyl-15-acetyl ester	1 μg/mL	0
64	20-Deoxyingenol	1 μg/mL	0
65	Aurantiamide	1 μg/mL	0
66	Aurantiamide acetate	1 μg/mL	0

hMSCs were treated with chemicals for 24 h, washed three times with medium, and cultured with hB cells for 72 h. CpG-oligodeoxynucleotide (5 μg/mL) was added to the culture of hB cells alone or to the co-culture of hB cells and hMSCs. The levels of IgM were determined by ELISA.

[a] Inhibition (%) = 100 × (1– B/A).

Table S2. siRNA sequences

Chemokine or receptor ligand	Sequences	GenBank accession number
Mouse CCL2	5′-GCUAAUGCAUCCACUACCUdTdT-3′<br>5′-UGAAGCUAAUGCAUCCACUdTdT-3′<br>5′-CACAACCACCUCAAGCACUdTdT-3′	NM 011333.1
Mouse CXCL12	5′-CUCCAAACUGUGCCCUUCAdTdT-3′<br>5′-CUGCAUCAGUGACGGUAA AdTdT-3′<br>5′-CAACGUCAAGCAUCUGAAAdTdT-3′	NM 001012477.1
Human CCL2	5′-CUCCGAAGACUUGAACACUdTdT-3′<br>5′-GCUCGCGAGCUAUAGAAGAdTdT-3′<br>5′-CUCACUCCACAACCCAAGAdTdT-3′	NM 002982.3
Human CXCL10	5′-GGUCACCAAAUCAGCUGCUdTdT-3′<br>5′-GAGAUCAUUGCUACAAUGAdTdT-3′<br>5′-CAUGAAUCAAACUGCCAUUdTdT-3′	NM 001565.3
Human CXCL12	5′-GAUUCUUCGAAAGCCAUGUdTdT-3′<br>5′-CCAGAGCCAACGUCAAGCAdTdT-3′<br>5′-CAACAGACAAGUGUGCAUUdTdT-3′	NM 000609.7
Human PD-L1	5′-CAGCAUUGGAACUUCUGAUdTdT-3′<br>5′-GAAUCAACACAACAACUAAdTdT-3′<br>5′-CUGACAUUCAUCUUCCGUUdTdT-3′	NM 001267706.1
Human FasL	5′-UCUUACCAGUGCUGAUCAUdTdT-3′<br>5′-CUCAGACGUUUUUCGGCUUdTdT-3′<br>5′-CUGAGCCACAAGGUCUACAdTdT-3′	NM 000639.2
Negative-control siRNA	5′-CCUACGCCACCAAUUUCGUdTdT-3′	

Table S3. Sequences of primers used for RT-PCR

Mouse	Sequences
CCL2	sense, 5′-GGAGAGACTATCAAGATAGT-3′
	antisense, 5′-ATGGTCAGTAGACTTTTACA-3′
CCL3	sense, 5′-CCCAATGAGTAGGCTGGAGA-3′
	antisense, 5′-GAACTGCCTTTGCCTTCTTG-3′
CCL4	sense, 5′-TCAGCGCCATGTGAGTCTAC-3′
	antisense, 5′-GGTTAGACCCTTCCACACCA-3′
CCL5	sense, 5′-CCCACTTCCTGCTGTTTCTC-3′
	antisense, 5′-CCCCATGACCTCACTGTTCT-3′
CCL19	sense, 5′-GCCAAGAGCAACAACTAGGC-3′
	antisense, 5′-ATTTGGAACCCAGCATTGAG-3′
CXCL9	sense, 5′-TCTCCTCCCTCCCCTTAGAA-3′
	antisense, 5′-CGGCTTTATTGGAAGCTCTG-3′
CXCL10	sense, 5′-CAGCAAGATGCCAGAAAACA-3′
	antisense, 5′-TGGCTGATCTGCAAGAAATG-3′
CXCL11	sense, 5′-GGATGGCTGTCCTAGCTCTG-3′
	antisense, 5′-ATAACCCCTTGGGAAGATGG-3′
CXCL12	sense, 5′-CAGTCCTCAATGCCTGTTCA-3′
	antisense, 5′-GAGCTTGTGGAGCATTGTCA-3′
CCR2	sense, 5′-CTTCATCCCCATTCTCCTCA-3′
	antisense, 5′-GACTCTGCTCTGGTGGAAGG-3′
CCR5	sense, 5′-GCTCATGATCCCTATCTCC-3
	antisense, 5′-CTGGGCACCTGATTTAAAGG-3′
CXCR4	sense, 5′-GCTGAAGAGCGTGACTGATA-3′
	antisense, 5′-GAGGACTGCATGTATAATGA-3′
COX2	sense, 5′-CCTTCCGAAGTTTCTGGCAGCAGC-3′
	antisense, 5′-GGCTGTCAGAGCCTCGTGGCTTTGG-3′
IL-1β	sense, 5′-GGCTGTAGAGCGAGTGTTGC-3′
	antisense, 5′-GTAGAGGTTGACAGTGTAGAT-3′
IL-12	sense, 5′-GAGTGTGGATCCCAAGCAAT-3′
	antisense, 5′-CTCAGTGCGGGCTATGACCA-3′
iNOS	sense, 5′-TGACGTCACTGGAGTTGTAC-3′
	antisense, 5′-GGTTCATGTCATGGATGGTGC-3′
IFN-γ	sense, 5′-GACTTGAAGATGTACCAGACAG-3′
	antisense, 5′-GAGATGAGATGTGATGGGAG-3′
TNF-α	sense, 5′-AGCGGCTGACTGAACTCAGATTGTAG-3′
	antisense, 5′-GTCACAGTTTTCAGCTGTATAGGG-3′
TGF-α	sense, 5′-ACGGCATGGATCTCAAAGAC-3′
	antisense, 5′-GGTCACTGTCCCAGCATCTT-3′
β-actin	sense, 5′-TGGAATCCTGTGGCATCCATGAAAC-3′
	antisense, 5′-TAAAACGCAGCTCAGTAACAGTCCG-3′

Human	
β-actin	sense, 5′-GTGGGGCGCCCCAGGCACCA-3′
	antisense, 5′-CTCCTTAATGTCACGCACGA-3′
TGF-α	sense, 5′-CTCGCCAGAGTGGTTATCTT-3′
	antisense, 5′-AGTGTGTTATCCCTGCTGTCA-3′
COX-2	sense, 5′-CCCGCCGCTGCGATGCTCGCCC-3′
	antisense, 5′-GACTTCTACAGTTCAGTCGAACG-3′
iNOS	sense, 5′-GCAGAATGTGACCATCATGG-3′
	antisense, 5′-ACAACCTTGGTGTTGAAGGC-3′
IDO	sense, 5′-CCATATTGATGAAGAAGTGGGCT-3′
	antisense, 5′-GATCAGGCAGATGTTTAGCAATGA-3′
CCL2	sense, 5′-ATGAAAGTCTCTGCCGCCCTTCTGT-3′
	antisense, 5′-AGTCTTCGGAGTTTGGGTTTGCTTG-3′
CCL3	sense, 5′-ATGCAGGTCTCCACTGCTGCCCTT-3′
	antisense, 5′-GCACTCAGCTCCAGGTCGCTGACAT-3′
CXCL10	sense, 5′-CCTGCTTCAAATATTTCCCT-3′
	antisense, 5′-CCTTCCTGTATGTGTTTGGA-3′
CXCL12	sense, 5′-ATGAACGCCAAGGTCGTGGTCG-3′
	antisense, 5′-TGTTGTTGTTCTTCAGCCG-3′
PD-L1	sense, 5′-TTGGGAAATGGAGGATAAGA -3′
	antisense, 5′-GGATGTGCCAGAGGTAGTTC-3′
PD-L2	sense, 5′-ACACCGTGAAAGAGCC-3′
	antisense, 5′-AATGTGAAGCAGCCAAG-3′
FasL	sense, 5′-GGATTGGGCCTGGGGATGTTTCA-3′
	antisense, 5′-TTGTGGCTCAGGGGCAGGTTGTTG-3′
ICAM-1	sense, 5′-CGTGCCGCACTGAACTGGAC-3′
	antisense, 5′-CCTCACACTTCACTGTCACC-3′
VCAM-1	sense, 5′-ATGACATGCTTGAGCCAGG-3′
	antisense, 5′-GTGTCTCCTTCTTTGACACT-3′
IL-1β	sense, 5′-ATGGCAGAAGTACCTAAGCTCGC-3′
	antisense, 5′-ACACAAATTGCATGGTGAAGTCAGTT-3′
IL-6	sense, 5′-AGACAGCCACTCACCTCTTCAG-3′
	antisense, 5′-TTCTGCCAGTGCCTCTTTGCTG-3′
IL-8	sense, 5′-CTTGGCAGCCTTCCTGATTT-3′
	antisense, 5′-CTTGGATACCACAGAGAATG-3′
IL-10	sense, 5′-ACCAAGACCCAGACATCAAG-3′
	antisense, 5′-GAGGTACAATAAGGTTTCTCAAG-3′
